# The role of clinicians in the looping effect: epistemic injustices and looping breaks

**DOI:** 10.1007/s11019-025-10279-2

**Published:** 2025-06-09

**Authors:** Christophe Gauld, Boris Nicolle, Axel Constant, Anne-Marie Gagné-Julien

**Affiliations:** 1 Service de Psychopathologie du Développement, Hospices Civils de Lyon, 69000 Lyon, France; 2 Institut Des Sciences Cognitives Marc Jeannerod, UMR 5229 CNRS & Université Claude Bernard Lyon 1, 69000 Lyon, France; 3 Service de Réhabilitation Psychosociale, Centre Hospitalier des Pyrénées, Pau, France; 4 School of Engineering and Informatics, The University of Sussex, Brighton, UK; 5 Department of Equity, Ethics and Policy, Centre for Research in Ethics (CRE), Canada Research Chair in Feminist Ethics (CREF), McGill University, Quebec, Canada

**Keywords:** Looping effect, Epistemic injustice, Experiential knowledge, Natural kinds, Credibility, Patient testimonial

## Abstract

The debate on whether psychiatric disorders can be studied as natural kinds has raised controversy, reviving socio-constructionist arguments about the influence of social factors on psychiatric categories. A key concept in this discussion is the “looping effect”, which describes how individuals change in response to their classifications, necessitating revisions to those classifications. We argue that, until now, the broad discussions around the looping effect have greatly failed to integrate the perspectives surrounding clinicians and patients. We examine more closely the dynamic and unstable nature of psychiatric diagnoses by proposing two key hypotheses: first, that understanding the looping effect requires incorporating both clinician and patient viewpoints, and that when done adequately, such an incorporation can facilitate the work of the clinician by creating feedback loops (i.e., the iterative adjustment of clinical interpretations based on patient responses); and second, that epistemic injustices between clinicians and patients can create disruptions in these feedback loops, which we call “looping breaks”, rendering them ineffective. Looping breaks can happen at the clinical level of the relationship between the patient and the clinician or at the nosological level (during the process of revising a classification). We suggest that looping breaks can be caused by a denial or minimization of credibility based on identity prejudice, or due to an epistemic disadvantage, affecting the experiential feedback of patients following the announcement of a diagnosis. To substantiate our claims, we first examine the impact of looping effects in the interaction between patients and clinicians. Second, we investigate the impact of these interactions at the nosological level, on the broader diagnostic framework. We identify epistemic injustices as critical factors that can lead to looping breaks at both levels, thus affecting the stability and validity of psychiatric diagnoses. Our findings underscore the importance of an epistemic approach to the looping effect, emphasizing both knowledge validity and justice in clinician-patient relationships and among clinicians themselves.

## Introduction

Whether psychiatric disorders can be studied based on natural kinds has given rise to intense controversy over the past decades. These controversies have notably revived a certain number of socio-constructionist arguments, i.e., on the influence of social factors on psychiatric categories. One of the most striking notions of this social influence on the definition of psychiatric disorders is the notion of “looping effect”, initially developed by Ian Hacking. This effect occurs in so-called “interactive” (psychiatric) categories, i.e., categories which are remodeled according to the behaviors of the people classified, behaviors themselves modified by their categorial designation (Hacking 1995a). According to this view, individuals change in response to how they are classified; if the classifications fail to accurately describe the testimonie(s) and behavior(s), then classifications are revised to do so.

In psychiatry, discussions about the implications of the looping effect have evolved in various directions. Some have focused on highlighting classification instability (Tsou 2007) and defining psychiatric kinds through this lens (Haslam 2016). Others have explored how these looping kinds can be particularly “capricious”, i.e., behaving in unexpected ways, contrary to existing theoretical understanding (Laimann 2020). Additionally, debates have been raised around issues of realism versus nominalism and essentialism versus constructionism as framed by this approach (Haslam and Ernst 2002; Khalidi 2010). There has also been criticism of the distinction between interactive kinds and indifferent kinds (Cooper 2004; Tsou 2007), and it was argued that Hacking’s account was incomplete because of a lack of specification regarding its conceptions of the self and of mental disorders (Tekin 2014, 2016). Except for some proposals in the context of complex systems theory and psychiatry (e.g., (Kirmayer and Sartorius 2007; Hauswald 2016; Constant et al. 2022; Gómez-Carrillo and Kirmayer 2023)), the literature has seldom discussed the impact of looping effect on the interaction between clinicians and patients or between clinical experts and nosology.

The aim of this article is to explore the critical role of clinicians in the process of nosological revision, arguing that the looping effect should be considered a beneficial mechanism rather than a source of instability, and emphasizing the importance of integrating epistemic justice to ensure the accuracy and fairness of psychiatric diagnoses. We ground our work in the framework of epistemic injustices. Epistemic injustices refer to the ways in which a person’s ability to produce knowledge can be unwittingly minimized or dismissed. This form of moral harm occurs when someone is unfairly judged as an unreliable knower (testimonial injustice) or is unable to contribute and access concepts that give meaning to their experience (hermeneutical injustice) due to negative identity prejudice (Fricker 2007).

Psychiatric practice seems predisposed to generating epistemic injustices. It is widely known that clinicians might not be fully able to receive or interpret certain essential information provided by patients, particularly regarding their subjective experience. And given that classifications are developed by clinical experts, they may be poorly reflecting (or lack of) systematic and comprehensive consideration of the experience and testimony of patients. Nosology may be “resistant” to modifications based on clinician or patient inputs (e.g., due to the absence of reception of experiential feedback). In parallel, groups of individuals designated by a diagnosis may lack the capacity to express their experiences effectively, fail to demonstrate their experiential knowledge, or lack the epistemic resources necessary to assert their testimonies and behaviors. These phenomena may take place through looping effect that operate at the level of the (unheard) patient and the (unreceptive) clinician—the “clinical level”, and at a “nosological level”, referring to the entire process of construction and revision of classifications.

The upshot of our exploration of the role of clinicians in the process of nosological revision is a map of the way epistemic injustices can harm patients through disrupting looping effects. We suggest in Sect. “Looping breaks and epistemic injustices” that there is an epistemic proximity between the notions of looping effect and epistemic injustice, through “looping breaks”, which we propose to define as barriers to revising a category caused by credibility denial or minimization due to identity prejudice, or by epistemic disadvantages affecting patient feedback. We do not claim to bring any refinement to the concept of epistemic injustice, which is only used here as a tool to better understand the notion of looping effect in clinical practice.

To develop this position, in Sect. “Looping effects in psychiatry”, we first address the tension between the need for nosological stability and the importance of epistemic justice, proposing to adopt a differential view between a clinical level of looping effects (considering forward and feedback looping effects between patients and clinicians) and a nosological level of looping effects (considering the bottom-up impact of clinicians, nosologists and communities on the revision of the categories). We consider how nosological revision operates through clinicians’ relation with their patients, highlighting the different temporal scales at which the looping effect operates, distinguishing between the short-term clinical interactions and the long-term nosological revisions, and examining how these different timescales impact the integration of patient feedback into diagnostic categories.

To summarize, this article explores the role of clinicians in nosological revisions through the lens of Hacking’s looping effect, distinguishing between clinical (i.e., interactions between patients and clinicians influencing diagnostic understanding) and nosological levels (i.e., the impact of clinical experts and patient testimonies on classification revisions). It argues that looping effects, in addition to potentially causing classification instability, contribute to refining psychiatric knowledge and improving diagnostic accuracy when epistemic justice is integrated. The concept of *looping breaks* is introduced to describe disruptions in these feedback loops due to epistemic injustices, particularly testimonial and hermeneutic injustices affecting patients. By analyzing the temporal scales of looping effects and the relationship between patient testimonies, clinicians, and classification revisions, this paper highlights the importance of epistemically just interactions in shaping psychiatric categories.

## Looping effects in psychiatry

Labelling theory emerged in sociology from the beginning of the twentieth century, under the influence of the work of Émile Durkheim, developed in particular by George Herbert Mead (Mead 1934), Edwin Lemert (Lemert 1951) or Erving Goffman (Goffman 2009). This theory states that the way people are classified, as well as the terms used, modify their behavior to align with the category. Thus, labelling theory posits that a diagnostic category can have an influence on individuals’ behaviors. However, contrary to labelling theory’s initial hypothesis, people seem to do more than merely trying to correspond to the stereotypes associated with the category to which they belong. Sometimes a classification can push individuals under that classification to modify their behaviour in response to the criteria established by the classification, which can ultimately require a reshaping of these criteria—a dynamic interaction described by Ian Hacking as the “looping effect”^1^ (Hacking 1995a, 1999). As human beings become aware of the categories by which they are classified (or “human kinds”, as Hacking calls it), they may come to modify their behaviours and conception of one-self (Khalidi 2010; Hauswald 2016; Vesterinen 2020). This means that the classification and the entity being classified interact dynamically.

Looping effects have been studied and discussed in several contexts (e.g., child abuse, women refugees, etc.—(Hacking 1995a, 2007)). In psychiatry, looping effects occur through the interaction with particular human kinds, namely diagnostic categories: people are labelled using official diagnoses, and these diagnoses subsequently shape how individuals understand, internalize, and express their condition. This evolving self-conception and behavior, as mediated by the diagnostic label, may in turn influence and ultimately contribute to the revision of the diagnostic category. One of Hacking’s classical examples is the diagnosis of multiple personality disorder (e.g., (Hacking 1992, 1995a, 1995b)). Hacking tells the story of what he calls the “epidemic” of multiple personality that began in the 1970s. Very briefly, a few cases of multiple personality became increasingly known to the public and clinicians through the publicization of “sensational cases” for which people showed different personalities amnesic to one another (e.g., via books, films, television shows and the media). A psychiatric label was then created to account for this phenomenon, namely “multiple personality disorder”. At first, one aspect of the clinical profile of these individuals was to present two to three personalities. Over time and with increased exposure to the diagnostic category, this typical profile evolved. Individuals began presenting with a significantly greater number of personalities, sometimes up to a hundred. Consequently, the symptomatology was adjusted to accommodate these new expressions of multiple personalities. Many psychiatrists also came to believe that childhood abuse was the primary cause of personality dissociation. As a result, treatments shifted focus towards uncovering patients’ memories of such abuse. Subsequently, patients started reporting experiences of childhood abuse (Hacking 1995a, 1995b, 2007).^2^

As illustrated with this example, diagnoses themselves are under the influence of the responses and behaviors of classified individuals. Thus, labelled individuals may come to understand and express themselves differently in light of their diagnosis, adjusting their testimonies and behaviors accordingly. This change in behavior can affect, by feedback, the category itself, and therefore official nosologies. In 2007, Hacking added more explicit components to the description of the way the looping effect operates, in offering what he calls “a framework analysis” (Hacking 2007). This framework would include five components:

I. A classification, detailing criteria (of “multiple personalities”, and later the diagnosis of “multiple personality disorder” in the Hacking’s example);
II. The people being classified, who are in the example at least unhappy, or unable to cope, or in distress;
III. Institutions, such as hospitals and the media (or “the International Society for the Study of Multiple Personality and Dissociation”);
IV. Knowledge, understood as “presumptions that are taught, disseminated, refined and applied within the context of the institutions” (Hacking 2007, p. 297) (like the knowledge that “early sexual abuse” was a causal factor in a large majority of cases of multiple personality);
V. The experts, who produce or legitimate the knowledge, and who assess its validity and use it in their daily practice (e.g., psychiatrists and psychologists who would try to help people with multiple personality disorder in eliciting memories of child abuse in the hope of mitigating its effect on dissociation) (Hacking 2007).

A first aspect that we want to highlight from this framework analysis is that the looping effect is a phenomenon which has an “epistemic life”, widely deployed within medical institutions and through the work of medical experts. The categories involved in the looping effect are, according to Hacking, categories that aim to accumulate knowledge about a group of classified people (Hacking 1995a). Through the looping effect, it is the *knowledge about a category of people* that is of interest: an expert community possesses this knowledge, which is validated and propagated by institutions, and which is modified according to the responses of classified people. The looping effect is therefore describing an epistemic process.

A second thing we want to stress is that for Hacking, the experts now play an explicit role in the looping effect, without which his analysis would be incomplete. By considering the experts as “key players” in the looping effect, Hacking raises new questions. First, how exactly do the experts (i.e., clinicians) influence the looping effect in psychiatry? Hacking does not develop more on this point. And what are the implications of understanding the looping effect as an epistemic process, given the role of the clinician in this interaction?

To start answering these questions, we will first suggest a distinction between two level at which clinicians have a role to play in the looping effect: the (individual) clinical level and the (population) nosological level. The first level involves incorporating a patient’s experiential knowledge in clinical practice, which can generate a looping effect. The second level involves using experiential knowledge from various patients during nosographic revisions, relying on clinicians to report patient testimonies and behaviors to update nosography—which can also generate a looping effect.

Thus, the looping effect *L* considering both the clinical and nosological levels can be summarized as follows: *L* = *f*(*C*_*r*_*, P*_*r*_) + *g*(*E*_*x*_*, N*), where *L* involves the clinician’s role (*C*_*r*_) and patients’ responses (*P*_*r*_) at the clinical level and includes the role of experts (*E*_*x*_) and the revisions of nosologies (*N*) at the nosological level. The equation *f*(*C*_*r*_*, P*_*r*_) represents thus the feedback looping effect at the clinical level, and the equation *g*(*E*_*x*_*, N*) represents the feedback looping effect at the nosological level.

This way of presenting looping effects enables us to demonstrate that, once the interactions between these different level-processes and the roles of the clinician are identified, the risks of epistemic injustice inherent in the looping effect become clearer. In the following sections, we will break down the looping effect into two components: first, we will analyze the aspect of the looping effect that occurs between the clinician and the patient; then, we will examine the component that takes place between experts and classifications.

### Looping effects at the clinical level

Patients’ testimonies and behaviors usually influence the category through the observation and collection of symptoms and narratives by clinicians. To illustrate this, we should consider here two dimensions of the looping effect: the “forward” aspect of the looping effect, which corresponds to the consequences on people of the way in which they have been labelled, and the “feedback” aspect of the looping effect, which corresponds to the consideration of behavioral and experiential changes in patients, by clinicians, to revise the category. Hacking himself speaks of this “feedback effect” in the context of child abuse (Hacking 1999), based on the idea of Li and colleagues who introduce the idea of the “feedforward cycle” (Li et al. 1990).

The forward looping effect occurs when a patient receives a diagnosis, meaning they are identified and labelled with a specific diagnostic category. It refers to the influence of a diagnostic label on a patient’s self-perception, testimonies, behaviors, and increasing awareness, leading them to identify with the label and modify their self-concept, behavior, and self-understanding in response to their classification. This identification is mainly done through the diagnosis during the clinical interview: here, the clinician is an intermediary protagonist between the nosography (which they learned during their training) and a patient (or the “people classified”, to use Hacking’s framework analysis). A large literature exploring clinical decision-making processes suggests that clinicians interpret diagnostic categories through the lens of their own clinical experience (Zarin and Earls 1993; Redelmeier et al. 2001; Bowen 2006; Croskerry 2009; Crumlish and Kelly 2009; Bhugra et al. 2010; Bhugra et al. 2011). Categories are therefore not used in the same way depending on different clinicians. For instance, the clinical specificities of each clinician correspond to their collection of symptoms in their clinical practice, to the specific labelling of these symptoms, to their theoretical care model, to the influence of guidelines and literature on their practice, to their personality, education and experience, their type of cognitive reasoning, and a whole set of “uncontrollable” factors (the limited time of the consultation, the external pressures of the institution, etc.) (Martin et al. 2022). These clinical specificities influence the categories that the clinician deploys daily. For example, a clinician trained in psychoanalysis will explore dimensions of the patient history, while a clinician trained in cognitive behavioral therapy will prefer to explore response patterns to concrete situations: their way of collecting symptoms will differ and will lead to potentially different diagnoses. Similarly, knowledge of guidelines can orient a clinician towards a different treatment from another clinician who is not aware of them. Therefore, the forward looping effect depends in part on these factors, i.e., on the complex clinical decision-making processes.^3^

At the clinical level, the feedback looping effect, denoted *f*, corresponds to the consideration of behavioral and experiential changes in patients by clinicians. In other words, it refers to the process by which clinicians adjust their diagnostic understanding based on patients’ behavioral and experiential changes. This looping effect is considered in the “brief” temporality of clinical practice, mediated by the clinician who diagnoses a patient, characterized by a short time frame, for example in weeks or months. Patients’ testimonies and behaviors do not directly impact the official nosology. Instead, this influence occurs indirectly through clinicians. While the formal diagnostic criteria (e.g., in the DSM) remain unchanged, clinicians’ perception of a diagnosis may be influenced by the ways in which patients come to understand, embody, and express their label.^4^ It can be denoted as *f*(*C*_*r*_*, P*_*r*_). Thus, a part of the feedback looping effect could be present at the clinical level even though the nosographies are not revised—this revision being however necessary, in a longer time, to speak of a loop effect. After receiving a diagnosis, these patients have developed a new awareness of themselves through the lens of the category, which has influenced their self-perception and actions. This specific response brings about a change in the way clinicians understand and use the category in their practice (Bhugra 2010; Bhugra et al. 2011). And this change in the clinician’s understanding and use of the category changes the way the diagnosis is communicated to patients—ultimately influencing how the clinician’s future patients interpret, integrate, and express their diagnosis. For example, autistic women without intellectual disabilities may exhibit social impairments that are challenging for an inexperienced clinician to recognize due to gendered socialization, potentially leading to the clinician overlooking their distress. After receiving a diagnosis, these patients have developed a new awareness of themselves through the lens of the category, which has influenced their self-perception and actions. They progress in their social understanding, allowing to better explain their difficulties to clinicians. In consequence, after having discussed with them, the clinician’s perception has also necessarily evolved (Zarin and Earls 1993; Redelmeier et al. 2001; Bowen 2006; Croskerry 2009; Crumlish and Kelly 2009; Bhugra et al. 2010, 2011). However, even if the perception of the women autism category has evolved for a given clinician, the category has not necessarily been revised according to their new perception.

### Looping effects at the nosological level

The looping effect can only be described because its two components, at the clinical and nosological levels, are present. At the nosological level, through the insights of clinical experts (who could be designated, more generally, as “health professionals”), diagnostic categories are revised based on how patients articulate, interpret, and manifest their diagnosis over time. For example, the attention deficit hyperactivity disorder (ADHD) category have been revised to include symptoms and specifiers that were not considered initially, such as subtypes, that appear since the DSM-IV (American Psychiatric Association 1994), or the possibility of a disorder before 12 years old and the notion of fluctuation of symptoms over time, which appear since the DSM-5 (American Psychiatric Association 2013). These changes might in part be explained because diagnosed patients have changed their behavior and their statements following the diagnosis, making them able to express themselves on particularities hitherto relatively invisible to clinicians.^5^

The exact way in which clinical experts “act” on nosographies needs to be more detailed. We are here interested in the feedback looping effect at the population nosological level, i.e., between clinical experts and the nosology, which happens when a set of clinical experts report a set of testimonies and behaviors—denoted by *g*. We define the feedback looping effect at the population nosological level as the process where clinical experts consider behavioral and experiential changes in patients to revise nosography, as represented by* g*(*E*_*x*_*, N*). This involves the collective influence of a community of clinical experts (“population”) on the revision of diagnostic categories (“nosological level”). Unlike the clinical level looping effect, this involves the impact of a group of clinical experts on potential nosological revision.

Many non-clinical arguments influence category changes. For example, the introduction of new validators for diagnostic categories is likely to alter the content of a category (Kendler 2013; Solomon and Kendler 2021; Solomon 2022). Similarly, an immeasurable set of political, economic, and industrial forces (Ghaemi 2009; Pickersgill 2014) influence classifications, as the APA cannot be entirely detached from the influences of various sources of pharmaceutical funding, professional interests, or regulatory considerations, which may constrain the extent to which concerns of epistemic justice are incorporated into classification changes (Cosgrove 2011). These forces are practically enacted by actors actively engaged in the loop of clinical and nosographic revision, as we will detail below. However, in this section, we focus on the (non-quantifiable) role of the clinical expert integrated into scientific review committees in the looping effect. This emphasis is justified by our primary interest in the clinician’s role within the nosological loops, an interest that should not negate the existence of other previously mentioned non-clinical factors. Two particularities of this feedback looping effect at a population nosological level can be noted. Changes in looping effect unfolds at different temporalities. It could be relatively “long”, e.g., years or decades. Indeed, in nosology, the revision of the classification criteria takes place over a relatively long-time interval, e.g., seven years between the DSM-III-R (Spitzer 1987) and the DSM-IV (American Psychiatric Association 2000) and nineteen years between DSM-IV and DSM-5 (American Psychiatric Association 2022). This time scale is important to consider. Indeed, in the brief temporality of clinical encounters, changes in looping effect are not noticeable, since they only take place when the feedback looping effect has been integrated into nosographic revisions—in a few years or decades by example. We will subsequently see how this dissection between time scales is of great importance for clinical practice, since some injustices can persist for years before the looping effect corrects them. Moreover, the category will not be changed due to the testimony or behavior of a single patient through a single clinician. Clinical experts aggregate a large set of clinical testimonies to sustain the revision of a classification system.

Two nuances should be provided to better understand the role of the clinical experts in scientific review committees. A first nuance must be provided regarding this feedback looping effect of clinical experts on nosology. In some cases, nosological revisions are made by individuals directly involved in the revision of psychiatric nosology (e.g., patients, their family, or members of an association, via lobbying or other forms of political actions). For instance, Kapp and Ne’eman trace their journey as autistic self-advocates who lobbied members of the DSM-5 Neurodevelopmental “Disorders” Working Group, who were responsible for revising the autism spectrum diagnostic, among others. This point raises the question of the representativeness and the weight given to these testimonies, which seems to remain relatively minor in the revision processes (Kapp and Ne’eman 2020). This nuance relating to the influence of patients, patient-researchers, experts by experience, resource persons and advisers, involved directly or indirectly in nosology revision, has been developed as a specific field in the scientific literature for more than thirty years (Marshall et al. 2001). This (too rare) involvement of stakeholders’ involvement (e.g., patients, families, advocacy groups—denoted as *S*) aligns precisely with the phenomenon we aim to illustrate here: in the case of autism for instance, individuals who are diagnosed respond to their diagnosis. They express dissatisfaction with the way autism is characterized in the DSM. This engagement, in turn, influences the diagnostic category through the lobbying efforts they employ during the DSM revision process. Consequently, the manner in which the category is described affects how diagnosed individuals will respond to their diagnosis in the future. For instance, the removal of the Asperger’s category from the DSM-5 results in individuals who would have been diagnosed under this category now being diagnosed within the broader autism spectrum. This shift impacts how they perceive themselves.

A second nuance could be provided concerning this feedback looping effect of clinical experts on nosology. The revision process is much more complex than simply collecting feedback from clinical experts. The shift from the clinical (individual) level to the population (collective) and thus structural level would require, in addressing epistemic injustices, considering the full range of individual forms of knowledge, including those of self-diagnosed individuals. However, it does not seem necessary to collect all such information; it is enough to consider the existing literature showing that mechanisms of epistemic injustice are necessarily widespread—particularly in the systematic dismissal by expert communities (researchers, clinicians) of testimonies from self-diagnosed individuals (Crichton et al. 2017).

For the sake of simplicity and related to the diversity of practices according to international classifications, we are talking about “nosological revision”, when we should speak of a “community of researchers” who participated in this revision, or of a “revision committee”. The complexity of the revision process involves a community of researchers and revision committees (denoted here as *C*). For example, the production of the ICD-10 (together with the construction of the DSM-IV) took about 10 years, from 1982 to 1992 (Üstün and Sartorius 1995), requiring the collaboration of nine work groups bringing together about 200 psychiatrists (during the 1982 Conference). Similarly, in 1974, the APA appointed 14 Nomenclature and Statistics Working Groups to begin work that would lead to the development of DSM-III. Each was considered an advisory committee, constituted of professionals with particular expertise in each psychiatric field (Demazeux 2013). This expertise allowed them to render a clinical opinion based on their observations and on the international literature, as described in the different versions of the DSM Sourcebook (i.e., the manuals collecting all the studies having sustained the constitution of the DSM categories) (Widiger 1998; Hester 2021).^6^ Within some of these work groups, expert patients and other people directly involved by psychiatry have been heard, particularly in the context of the constitution of the DSM-5 from 2002 to 2013 (Kapp and Ne’eman 2020, p. 5). Identifying the factors related to the selection of studies that led to the diagnostic categories and their revisions would require historical work that goes far beyond the scope of this research. Moreover, our aim is not to discuss the development of a specific classification in a narrow sense (e.g., the writing process within the APA), but in a broader way, including the possible range of knowledge, actors, and contextual factors that directly or indirectly influence a nosological production. In this broader view, the arguments put forward about the DSM could also apply to other classifications, in different contexts, past or future. However, if the looping effect at the nosological level considers formal and informal research results, it depends on the relationship between research investigators and study participants, further complicating the looping effect process. It is indeed through their research work that clinical experts can report their hypotheses and daily clinical observations.

Thus, the feedback looping effect at the nosological level, involving experts’ roles and nosological revisions and denoted as *g*(*E*_*x*_*, N*), should more precisely weight *S* (for stakeholders) and *C* (for the community of researchers) such that *g*(*E*_*x*_, *N*, *S*, *C*). We demonstrated the important role of clinical experts in understanding the looping effect in psychiatry, both at the individual and population levels. In the following sections, we will emphasize the importance of patient testimonials and behaviors in relation to the clinician’s role in the looping effect. This will highlight that clinical experts can cause looping breaks, leading to epistemic injustices. We will argue that it is not necessarily the category itself that hinders the looping effect and classification revision (Hacking 1999; Tsou 2017), but rather the patient’s inability to be heard due to negative identity prejudice and resulting epistemic injustices.

### Risks and benefits of looping effects in psychiatry

The looping effect, denoted as *L*, involves the feedback loop between patients’ responses (*P*) to their diagnostic category (*D*) and the subsequent modification of that category. This can be expressed as *L*(*P,D*). This process should be understood as an epistemic process (*E*), where clinicians and clinical experts (*E*_*x*_) play an important role represented by *E*(*E*_*x*_) (Table 1).

**Table 1:** Risks and benefits of looping effects in psychiatric diagnosis

	Interaction (locus)	Risks in case of looping break	Benefits
Clinical loop	Clinician—patient	Clinical mismatch: If the looping effect is suppressed, an incompatibility arises between the clinician’s diagnostic approach and the patient’s evolving state	Recognizing the moving ontology of the patient allows adaptive treatment and refinement of the diagnostic category Looping effects help capture the dynamic nature of the patient’s situation, allowing the clinician to interpret the diagnostic category under consideration
Nosological loop	Clinical experts/community—classification	Knowledge mismatch: Without appropriate revision, diagnostic classifications fail to capture the full spectrum of disorders, creating a mismatch between established classifications and clinical observations	Diagnostic categories evolve through population-level feedback, refining classifications Stabilizing a diagnostic category allows for better treatment adherence when the category resonates with the population (e.g., “depression as a serotonin disorder”)

Each loop demonstrates how interactions either between the clinician and patient or between clinical experts and diagnostic categories can result in harmful conflicts (testimonial or hermeneutic injustices leading to looping breaks)

Contrary to the view that the looping effect leads to classification instability, denoted as *C*_*i*_, we argue that under certain conditions and for specific psychiatric diagnoses, represented by (*B*), the looping effect can be beneficial. This beneficial impact (*B*) is particularly useful for dynamically revising psychiatric classifications, expressed as *R*(*T*_*p*_,* B*_*p*_), which considers patients’ testimonies (*T*_*p*_) and behaviors (*B*_*p*_). The formula: *L*(*P*, *D*) = *E*(*E*_*x*_) + *B* ⋅ *R*(*T*_*p*_*, B*_*p*_) − *C*_*i*_ captures the idea that the looping effect, guided by clinical experts’ epistemic processes, can have a beneficial impact under certain conditions, leading to the dynamic revision of psychiatric classifications by incorporating patients’ feedback.

In other words, we argue that, under certain conditions and for certain psychiatric diagnoses, it may prove particularly beneficial for dynamically enriching psychiatry. This effect is indeed particularly useful for properly revising psychiatric classifications, by considering the patients’ testimonies and behaviors. It is already acknowledged that diagnoses can be useful in current clinical practice (e.g., to guide patients care); but considering how patients respond to a diagnosis can also be very useful. A patient’s response to their diagnostic category can be interpreted as convenient sources of information that could improve the classifications in the long term.

For instance, the diagnosis of major depressive disorder (MDD) can significantly impact both the patient and the clinician’s approach to care. Upon learning that their symptoms are part of a recognized condition such as MDD, a patient may experience a reduction in self-blame and stigma, which can lead to adaptive psychological and behavioral changes, resulting in new combinations of MDD symptoms in this same patient. These shifts allow the clinician to adjust quickly to this individual case, considering the new patient’s specific symptom combination, which differs from their original presentation of MDD. Thus, the diagnosis pushed the clinician to refine their decision-making process in response to this reconfiguration of MDD symptoms, thereby creating a looping effect at the clinical level which proves to be positive for the patient’s care. We now turn to a discussion of the concept of “looping breaks” that can occur, as we will argue, because of epistemic injustices.

## Looping breaks and epistemic injustices

### Epistemic injustices in looping effects

Epistemic injustice refers to a specific class of harm in which a person’s ability to position themselves as an epistemic subject, i.e., producer/sharer and/or user of knowledge, is unwittingly denied or diminished due to negative identity bias or a systemic marginalization (Fricker 2007). The framework of epistemic injustice explores from an ethical and political point of view the ability of people to produce knowledge and the ability of their interlocutor to consider it. In philosophy of psychiatry, it has been argued that psychiatric patients are especially vulnerable to epistemic injustices in health care contexts (Sanati and Kyratsous 2015; Crichton et al. 2017; Kurs and Grinshpoon 2018). Two kinds of epistemic injustices have been traditionally identified and described: testimonial and hermeneutical injustices (but see (Kidd et al. 2022) for a comprehensive review).

Testimonial injustices are based on the existence of negative prejudices unfairly diminishing the credibility of the individuals. An individual’s ability to share knowledge can depend on their perceived credibility within a community. When individuals are deemed to have little credibility, their testimony is often ignored, treated with suspicion, or unsolicited. While a lack of credibility may be justified in cases like known liars, it becomes unjust when based on negative identity prejudices associated to marginalized social groups. Such unfounded biases, related to ethnicity, gender, sexual orientation, or social class, lead to testimonial injustice. According to Fricker, this occurs when a speaker is unfairly discredited, not due to actual lack of credibility, but because of prejudiced assumptions, thereby diminishing their potential for epistemic contribution—leading to testimonial injustice (Fricker 2007). Testimonial injustices are suffered by people having received a psychiatric diagnosis (Sanati and Kyratsous 2015; Crichton et al. 2017; Kurs and Grinshpoon 2018; Kidd 2019). Some have argued that negative prejudices about people with psychiatric diagnoses could be more widespread and more entrenched than those about several other pathologies (e.g., Carel and Kidd 2014). Stigma, social isolation, professional inactivity, or cognitive disabilities that affect patients (Thornicroft et al. 2007; Varshney et al. 2016) can all lead to a devaluation of their credibility (Crichton et al. 2017). Moreover, certain psychiatric symptoms make individuals especially susceptible to experience such testimonial injustices (Newbigging and Ridley 2018). For instance, the difficulty in distinguishing a substantiated allegation from delusional speech, potentially associated with symptoms of psychosis, can lead to a credibility deficit (e.g., the non-management of a somatic symptom such as chest pain, because it is confused with a somatic delusion, see (Kurs and Grinshpoon 2018) for similar examples).^7^

An hermeneutic injustice occurs when an individual’s epistemic abilities are undermined due to a lack of necessary interpretative resources to make sense of an experience (Fricker 2006). Hermeneutical injustice designates the cognitive disadvantage suffered by an individual when trying to interpret a social experience for which they, their community, or dominant circles have an incomplete understanding. It generally arises from a gap in conceptual resources for analyzing and interpreting an experience (Newbigging and Ridley 2018) and is rooted in hermeneutical marginalization, where an individual is denied full participation in understanding a wide range of social experiences (Fricker 2006). In psychiatry, this type of injustice mostly affects people who have received a psychiatric diagnosis, understood as a marginalized group. Diagnosed marginalized individuals may lack interpretive resources to make sense of their experience (Fricker 2007; Carel and Kidd 2014) or may have to use incomplete or problematic hermeneutic resources to be able to communicate their experience to others, such as pejorative terminology found in official nosography (Bueter 2019) or inadequate frameworks to understand distress or difference (Chapman and Carel 2022; Gagné-Julien 2022). Think for example of a dyslexic child in a rural community where dyslexia is not recognized due to the exclusion of neurodivergent individuals from educational discourse. The child’s behavior may be misinterpreted by teachers as signs of “laziness” or “lack of intelligence.” Only when the concept of “dyslexia” is properly explained to the child, family, and teachers, might the child’s disabilities be accurately understood and no longer subject to discrimination. Initially, the child could lack the hermeneutic resources to articulate their experience, and this community’s lack of these resources results in a hermeneutical disadvantage. While both teachers and the child share this hermeneutical disadvantage, the notion of injustice implies unfair harm suffered by the child. Thus, the dyslexic child is the victim of epistemic marginalization, unable to access the resources to make sense of their experience.

In both testimonial and hermeneutical injustice, it is important to stress that it is precisely because patients can bring decisive information about their diagnosis (Wylie 2014) that they are often victims of epistemic injustice. They have knowledge to share, but because of an unfair lack of credibility, this knowledge is not considered. For instance, they can draw attention to symptoms usually not considered by official nosology (Bueter 2019). They can talk about the potential benefits and threats of a diagnostic revisions, or informing experts about the impact of nomenclature (e.g., Bueter 2019; Gagné-Julien 2021). They can shed light on the positive value of a “pathological” experience that might be excluded by the traditional diagnostic manuals (Scrutton 2017). In addition to harming patients as epistemic agents, ignoring this information would cause epistemic losses (Drożdżowicz 2021).

Both testimonial and hermeneutical injustices can occur during the looping effect process. As described in Sect. “Introduction”, the looping effect involves an uptake of patients’ response to their diagnostic category, which in turn can modify the category. This, we have emphasized, should be understood as an epistemic process, in which the clinical experts have a key role to play.

### Looping breaks

One of the rationales of this article is to insist on the importance of the clinician and the patient’s response to categories in the debate on psychiatric kinds. However, how patients relate to a category may not be properly considered by clinicians. We suggest that epistemic injustices can cause “breaks” in the looping effect. A “break” in the looping effect participates in the unintentional ignorance, minimization, or denial of how patients engage with their diagnosis, even when such engagement could provide a useful source of information. This, we claim, constitutes a testimonial injustice. We thus define looping breaks as obstacles impeding the revision of a category, due to an unfair denial or minimization of patients’ credibility based on identity prejudice. This lack of consideration of patient’s acceptation and awareness to their diagnostic categories can in turn lead to a hermeneutical injustice, in that the knowledge produced about a diagnostic category will tend to be incomplete or harmful. Thus, based on the epistemic process driven by clinicians and clinical experts (*E*_*x*_), represented by *E*(*E*_*x*_), we denote epistemic injustices as *E*_*i*_ and we introduce the concept of looping break as *L*_*b*_. This concept considers both the clinician’s role, *C*_*r*_, and patients’ responses *R*_*p*_, as essential sources of information. The formula *E*_*i*_ = *L*_*b*_(*C*_*r*_, *R*_*p*_) illustrates that to address epistemic injustices effectively, we need to consider a looping break that incorporates both the clinician’s role and patients’ responses as core components. Thus, the looping effect process can be enhanced by addressing epistemic injustices through the concept of a looping break, as represented by *L*(*P*, *D*) = *E*(*E*_*x*_) + *B* ⋅ *R*(*T*_*p*_*, B*_*p*_) − *C*_*i*_ − *E*_*i*_.^8^

In order to explain how patient feedback can influence nosological revisions, we have further detailed how such feedback is necessarily filtered through clinical “actors”, whose role consists in aggregating and translating individual experiences. This process does not deny the existence of alternative explanations for the forces shaping classification revisions (e.g., the construction of societal values may not always occur through relational networks), but it avoids reducing epistemic change to purely sociological or constructivist dynamics. We stress the need to better account for potential hierarchical asymmetries in these chains of transmission. We argue that clinicians often act as collaborative spokespersons, mediating patient experience within institutional or political arenas (Latour 1993). We therefore complement the epistemic value of patient testimony with a normative constraint: the view that patient experience should serve as the foundational basis for any legitimate political, administrative or economic influence when it comes to revising psychiatric categories.

This value-oriented stance does not deny the existence of non-clinical influences, which are often difficult to affect through such testimonies. For instance, pharmaceutical lobbying may have played a role in the introduction of Disruptive Mood Dysregulation Disorder (DMDD), expanding the fuzzy boundaries of bipolar disorder and potentially allowing for new medication justifications under regulatory and legal pressure (Frances 2014). However, our argument does not seek to provide a definitive assessment of the relative impact of these influences. Rather, this minimally normative position aimed at reinforcing the centrality of the patient’s voice in the feedback loop. Such a perspective is pragmatically useful in that it encourages clinicians to actively participate in preventing looping breaks, by assuming the role of epistemic mediators committed to a shared deliberative process with the patient.

As such, we also acknowledge that patient feedback cannot be taken at face value without critical examination. Participatory processes require safeguards against potential distortions (e.g., self-diagnosis driven by non-clinical motivations—(David and Deeley 2024)). Avoiding looping breaks thus involves treating the patient as a co-investigator: questioning, discussing, and jointly interpreting their experience. This requires encouraging equal epistemic participation, allowing for negotiation and compromise between clinician and patient (Sakakibara 2023; Broeker and Arnaud 2024).

**Fig. 1:**
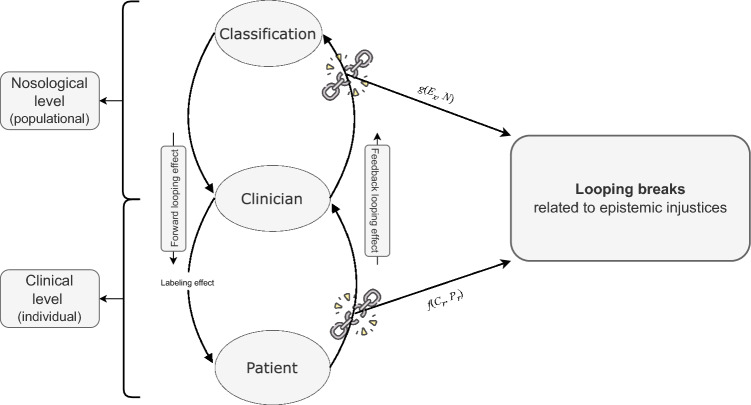
The roles of the clinician, the patient and the nosology related to the looping break. The figure illustrates the complex dynamics involved in the interactions between clinicians, patients, and diagnostic classifications. The clinical loop (involving the interaction between clinician and patient) and the nosological loop (involving the interaction between clinical experts and diagnostic classifications) offer opportunities for constructive feedback, but they also pose risks of epistemic injustices, which can lead to looping breaks. The looping effect *L* summarized as: *L* = *f*(*C*_*r*_, *P*_*r*_) + *g*(*E*_*x*_*, N*), where *f*(*C*_*r*_*, P*_*r*_) represents the feedback loop at the clinical level involving the clinician’s role (*C*_*r*_) and patients’ responses (*P*_*r*_), and g(*E*_*x*_*, N*) represents the feedback loop at the nosological level involving experts (*E*_*x*_) and nosology revisions (*N*)

Thus, in clinical terms, looping breaks can indeed be caused by testimonial and hermeneutic epistemic injustices. When clinicians commit testimonial injustices against patients when patients are responding to the diagnosis, it disrupts the looping effect. Given that diagnostic categories are always defined within specific sociocultural contexts, there can never be a singular “perfect” understanding that eliminates the looping effect: this means looping breaks can occur in any diagnostic process.^9^ Looping breaks can occur at the clinical level (i.e., between a patient and a clinician—denoted as *f*(*C*_*r*_*, P*_*r*_)) and/or at the nosological level (i.e., between clinical experts and nosology—denoted as *g*(*E*_*x*_*, N, S, C*)). The functions *f* and *g* could therefore be integrated into the equation *L*(*P*, *D*) = […] + *f*(*C*_*r*_*, P*_*r*_) + *g*(*E*_*x*_*, N, S, C*). The relationships between the looping breaks and the place of the clinician, the patient and the nosology are provided in Fig. 1. By recognizing and addressing epistemic injustices, psychiatry can mitigate the risks of looping breaks.

In the clinical loop, the patient’s evolving feedback—reflecting behavioral changes and self-awareness in response to the effects of their diagnosis—should guide the clinician in refining their diagnosis and treatment approach. However, when this feedback is ignored or dismissed, testimonial injustice occurs, preventing the clinician from fully recognizing the patient’s needs (themselves directly related to how they are adapting to the changes associated with their diagnostic label). This dismissal results in a clinical mismatch, where the patient’s evolving condition no longer aligns with the clinician’s diagnostic approach. For example, a patient diagnosed with bipolar disorder might recognize their own early warning signals of a manic episode (e.g., increased irritability or impulsivity), as initially explained to them by the clinician, but if the clinician dismisses these insights, the opportunity to adjust the treatment plan and prevent escalation could be missed. This situation creates a looping break, as the clinician’s dismissal of the patient’s insights prevents any consideration of the modified or newly emerging manifestations of the condition. By disregarding the patient’s evolving understanding of their symptoms, the clinician fails to update the diagnostic and treatment approach, leading to a potential stagnation in care that no longer aligns with the patient’s current experience of their disorder. This stagnation is further intensified when clinicians fail to create an environment in which the patient feels acknowledged as a credible epistemic agent. Beyond symptom recognition, effective clinical engagement relies on the capacity to actively listen, to approach testimony without prejudice. Welcoming the patient’s words as meaningful contributions supports the co-construction of a shared interpretive framework: this attitude requires clinicians to accept the limits of their own epistemic perspective and to adopt a stance of epistemic humility: a readiness to question assumptions, remain open to alternative narratives, and engage in a reflective and responsive therapeutic alliance (Gosselin 2018; Lane 2020; Stein et al. 2024). When this process is disrupted, whether through skepticism, misinterpretation, or an unwillingness to integrate the patient’s perspective, a looping break can occur. It not only reinforces testimonial injustice but also erodes the trust and openness necessary for meaningful clinical dialogue.

Additionally, hermeneutic injustices can arise when clinicians rigidly adhere to established diagnostic criteria without acknowledging or integrating the patient’s capacity to interpret their own experiences in light of their diagnosis. The patient is denied access to the interpretative resources necessary to fully understand or express their condition. As a result, the patient suffers a cognitive disadvantage, unable to participate in shaping the meaning of their experience. In psychiatry, this marginalization leads to a gap in conceptual resources, preventing both the clinician and the patient from adapting the diagnostic understanding to the patient’s lived experience, and reinforcing the disempowerment of the diagnosed individual. This results in a looping break, where the failure to integrate the patient’s evolving interpretations restrains the refinement of the diagnostic approach, perpetuating a misalignment between the patient’s condition and the clinical framework. This tension reflects the double bind characteristic of politically charged medical categories: while diagnosis can constrain a patient’s trajectory through excessive pathologization, it can also function as a vital epistemic resource—that is compromised when diagnostic labels are appropriated and lose their clinical significance, thereby denying patients the conceptual tools needed to articulate their experience and hindering the refinement of diagnostic understanding through careful, non-reductive medicalization (Spencer and Carel 2021). Such conceptualizations, adjusted for the patient, the clinician, and the medical system, require both conceptual analysis and engineering (Isaac et al. 2022), as well as a clarification of the underlying assumptions necessary for theorization (Maatman 2021).

The nosological loop emphasizes the need for diagnostic categories to evolve in response to population-level feedback. When psychiatric classifications remain static and fail to incorporate nuanced patient behaviors, a knowledge mismatch emerges. This stagnation in diagnostic refinement leads to a looping break at the nosological level, driven by both testimonial and hermeneutic injustices. These breaks occur when the input from the broader clinical community is undervalued, preventing diagnostic categories from evolving in response to new insights and lived experiences (Table 2).

**Table 2:** Prevention of looping effects caused by epistemic injustices in psychiatric diagnosis classifications

	Cause of looping break	Preventing harm
Clinical loop	Testimonial: Ignoring the patient’s interpretative capacity and feedback prevents the recognition of evolving patient needs	Leverage mechanisms of clinical listening and eliciting responses through patient engagement (e.g., empowerment)
	Hermeneutic: Clinician’s rigid adherence to established diagnostic criteria leads to a looping break by failing to acknowledge the patient’s interpretive capacity and the dynamic nature of their lived experience	Incorporate the patient’s interpretative resources and evolving self-understanding into clinical practice to prevent hermeneutic injustice and ensure a more adaptive diagnostic approach
Nosological loop	Hermeneutic: Diagnostic classifications fail to capture the nuances of disease classes, leading to stagnation in nosology	Leverage diagnostic listening by revising criteria based on population feedback. Use diagnostic eliciting to assess societal response to new classifications
	Testimonial: The dismissal of community input leads to a looping break in classification evolution	Regularly revise classifications by integrating experiential and societal feedback to avoid hermeneutic injustice

In the context of individual clinical practice, particularly regarding testimonial injustices, we argue that psychiatric patients are at significant risk of being subjected to recurrent epistemic injustices. For example, consider a patient diagnosed with MDD. Initially, the diagnosis concerns the patient, but it also provides a framework for understanding their symptoms and seeking help. The patient begins engaging in various therapeutic activities, such as attending therapy sessions, participating in support groups, and adhering to a medication regimen. These proactive steps lead to significant improvements in the patient’s mood and overall well-being. However, despite these positive changes, the patient may experience guilt related to the diagnosis itself, which is often stigmatized. The clinician, relying heavily on the established diagnostic criteria for MDD, might overlook this guilt stemming from the diagnosis. This neglected awareness could have provided an opportunity to refine the therapeutic approach. The clinician’s failure to consider the patient’s additional concerns results in a testimonial injustice. When this rejection occurs systematically across many clinicians, it could hinder the long-term revision of the category of MDD itself. By ignoring the changes in response to the diagnostic process, clinicians may contribute to a stagnant understanding of this disorder. This lack of consideration prevents the psychiatric community to update and refine the diagnostic criteria, and thus treatment protocols, for MDD. This dismissal creates a looping break.

Looping breaks may also stem from hermeneutic injustices. The broader understanding of MDD remains incomplete when neither clinicians nor diagnostic classifications manage to acknowledge, integrate, or utilize the concepts brought forth by patients following the disclosure of their diagnosis.

In summary, although the initial diagnosis of MDD may have positively influenced the patient’s behavior, leading to significant improvements, the clinician’s failure to account for the patient’s ongoing feedback can create looping breaks. These breaks impede both the immediate adaptation of treatment and the long-term evolution of the diagnostic category.

## Conclusion

We have analyzed the intricate relationship between the looping effect and epistemic injustices in clinical psychiatry. By differentiating from a large part of the literature on the looping effect, this article emphasizes the essential roles of clinicians and patients in the dynamic process of nosological revision, and how their interactions can lead to “looping breaks”. Integrating epistemic justice into clinical practice and nosological revisions can help mitigate looping breaks, ensuring that patient feedback is adequately considered. This approach balances the need for nosological stability with the imperative for justice, promoting a more inclusive and responsive psychiatric practice.

Such an approach, of course, requires further refinement of the multiple socio-economic and structural influences that have simultaneously shaped the stabilization and attempted refinement of psychiatric classifications (Ghaemi 2009; Cosgrove 2011).

Such analysis of the looping effect and epistemic injustices seems important for at least four reasons. First, the relationships between interactive psychiatric kinds, clinicians, and epistemic injustice, had been understudied. Given the importance of the patient experiential knowledge in contemporary debates in (philosophy of) medicine, the notion of looping break for thinking about the intertwining between testimonies and behaviors of patients, clinicians, and the nosology could constitute a philosophical tool particularly useful. The knowledge of this notion by concerned individuals can be beneficial with the aim of improving care, especially by counter epistemic injustice. This means that knowledge of the different parameters of the looping effect and looping break should ideally result in the negation or elimination of epistemic injustices, such as *L*(*P*, *D*) = *E*(*E*_*x*_) + *B* ⋅ *R*(*T*_*p*_*, B*_*p*_) − *C*_*i*_ − *E*_*i*_ [+ *f*(*C*_*r*_*, P*_*r*_) + *g*(*E*_*x*_*, N, S, C*)] ⇒ ¬(*E*_*i*_). Secondly, by placing the clinician and the patient at the center of our analysis, we enable them to have a better understanding of these different concepts, which could ideally support better care. Indeed, the absence of knowledge of a concept by clinicians prevents them from offering sustained listening and attention to their patients. Conversely, knowledge of the relations between notions of looping effect, epistemic injustice—and their potential looping breaks—constitutes the best way to avoid disruptions in care. Importantly, improving feedback loops requires not only recognizing their existence but also actively working to ensure that patient testimonies are meaningfully incorporated into both individual clinical practices and broader nosological revisions. Thirdly, the looping effect is usually perceived as a threat to the validity of diagnostic categories, presented as a classification instability operator. In this article, we argued instead that the looping effect contribute, in some way, to improving knowledge of psychiatric diagnoses and to promoting the patient interests. Ensuring epistemically just interactions between patients and clinicians allows feedback loops to support the refinement of psychiatric classification. Properly acknowledging the changes patients exhibit following the announcement of their diagnosis is an ethical imperative. Finally, we have identified two key areas where the looping effect operates: clinical and nosological levels. At the clinical level, clinician interpretations of diagnostic categories are influenced by patient testimonies and behaviors, often affected by epistemic injustices. This feedback looping effect shapes the clinician’s understanding of diagnoses. At the nosological level, collective clinical expert inputs can revise diagnostic categories over time. However, epistemic injustices can still cause looping breaks, where marginalized patient feedback are ignored, perpetuating inaccuracies and injustices in psychiatric classifications. Ensuring that feedback loops remain functional and effective requires addressing these barriers through systematic changes in both clinical and nosological practices. These three points have thus helped us to demonstrate the important role of clinicians in the process of nosological revision, arguing that the looping effect should be considered a beneficial mechanism rather than a source of instability, and emphasizing the importance of integrating epistemic justice to ensure the accuracy and fairness of psychiatric diagnoses.

## Data Availability

Not applicable.

## Abbreviations

ADHD: Attention deficit/hyperactivity disorder
BD: Bipolar disorder
DMDD: Disruptive mood dysregulation disorder
DSM: Diagnostic and statistical manual
ICD: International classification of disease
MDD: Major depressive disorder

## Footnotes

1 Hacking’s analysis of diagnostic categories is rooted in the metaphysical tradition and the philosophy of language concerned with natural kinds, and more particularly with psychiatric kinds (Zachar 2015; Tsou 2016; Tabb 2019). Hacking’s thesis on psychiatric kinds contains two versions (Forest and Faucher 2021). In a first version of his thesis, in 1995, Hacking opposes natural kinds and “human kinds”, the first being “indifferent” and the second being “interactive” with the way in which they are designated (Hacking 1995a). In a second version of his thesis, in 1999, Hacking asserts that the natural kinds themselves cease to be necessarily indifferent, since there could be an interaction between a disorder based in part on biological elements (with a biolooping effect) and a modifiable behavior through labelling (Hacking 1999). We tend to agree with this second version and the distinction between the indifferent part and the interactive part of the psychiatric categories. The psychiatric diagnostic categories would indeed include an indifferent part and an interactive part, sensible to the influence of individual experiences, representations of society and cultures (Tsou 2007). In this paper however, this distinction does not play a major role in our argumentation so we will leave this aspect of debate aside.

2 Since at least the DSM-III, while the evolution of diagnostic criteria is undeniable for certain disorders, such as autism, which has showed significant intra-spectrum variability following the broadening of its criteria (Rabot et al. 2023), several other diagnostic categories have remained impermeable to personal narratives and testimonies, and likely also to various political, social, and economic pressures (Moran 2021). There is, therefore, a form of prudential conservatism that reflects public health considerations, extending beyond the potential looping effects that could have influenced the categories (Demazeux 2015)—we will return to this point in the final section of the article.

3 This forward looping effect can be related to a literature on the performative aspect present in the dual relationship between caregivers and patients (Austin 1975; Loxley 2006; Butler 2014). Performativity refers to the fact that the words “do something”, they are likely to modify the relationship, the representations, and have a direct action on the psychiatric category. For instance, when the patient is questioned, her role is then to answer. Thus, the assignment of her diagnosis constitutes a relational commitment with the clinician. The clinician, whose understanding of categories is shaped by her clinical perspective, will influence her patient. Note, however, that labelling is not necessarily mediated by the clinician: it can be directly operated via a self-diagnosis, or partially mediated by the clinician when the patient has recourse to other sources of information (e.g., with the dissemination of the DSM criteria and the greater awareness of mental health in the general population, it often happens that parents seek confirmation of disorder for their child, or that they recognize in their child the same emergent disorder that they had experienced in their youth).

4 Based on this reading, it is important to note that a label does not appear to be intrinsically positive or negative (e.g., schizophrenia would not be necessarily stigmatizing, nor would attention deficit hyperactivity disorder necessarily be emancipatory or provide a way to name a disability), but rather depends on the patient’s interpretation. This interpretation is shaped precisely through contact with a clinician: if the label enables the patient to sustain a productive clinical relationship, forming an internal loop between clinician and patient, it is likely to be interpreted positively—and conversely. From the clinician’s perspective, when they acknowledge that they are using fluid categorizations, both the patient and the label may change; this recognition of influence can be used by the clinician to sustain the relationship and by the patient to better appropriate the diagnosis.

5 Our proposal concerns both the institutional practice of nosological revision and the clinical practice of diagnosis. While a better understanding of the effect of epistemic injustices in looping effects at the level of the clinical rapport is unlikely to have a direct effect on the institutional process of nosological revision, it could help reshaping diagnostic practices. Indirectly, through reshaping practitioners’ understanding of looping effects, our proposal may “bubble up” (Dennett 2009), as it were to come to influence how decisions are made at the institutional level. Indeed, any professional organization, including the APA, is made of communities of professionals who receive an education shaped by theoretical knowledge, and whose understanding of issues in psychiatry will come to shape institutional decisions. We believe that reshaping psychiatry involves both indirect, theory driven effort aimed at addressing issues of epistemic injustices in psychiatry and direct political efforts acting at the institutional level. Our proposal is an effort of the former kind. The clinician is indeed a central intermediary most of the time, disseminating patients’ perspectives to the broader community—which does not deny the significant existence of more direct links between patients and nosological changes (e.g., between self-diagnosed groups and the research community).

6 For the DSM-5, the web pages of the justifications and academic sources of the diagnostic criteria are accessible on the Internet Archive: https://web.archive.org/web/20220000000000*/DSM5.org. These are invaluable primary sources for understanding how the incorporation of clinical observations, through research, has led to changes in diagnostic criteria.

7 However, any disregard for the patient does not create such injustices. Failure to consider the patient’s knowledge does not constitute testimonial injustice per se. Testimonial epistemic injustice requires the existence of negative prejudices against the patient. Clinicians can deliberately not take into consideration (part of) the patient’s discourse, e.g., because they consider that the diagnosis could cause significant psychic distress and/or prevent the search for a differential diagnosis. Or they could possess evidence that the patient is self-deceptive or is lying. Moreover, considering some of the symptoms reported by the patient as not integral to the diagnosis is not necessarily a sign of negative bias: it may reflect the heterogeneity of a psychiatric disorder. In this way, questioning the patient’s narrative may be a way for the clinician to avoid a diagnostic error. For example, in a situation of conflict of loyalty and instrumentalization of an adolescent between her two divorced parents, the narrative of this adolescent may constitute the reflection of a necessary allegiance to one of the parents, which should however be qualified: the adolescent’s narrative should not be taken at face value, otherwise the conflict situation and the adolescent’s mental health could worsen. Thus, when the ignorance of the patient’s narrative is voluntary, and based on good reasons, it should not systematically be considered as epistemically prejudicial and ethically harmful.

8 Just as looping effects can be influenced by various socio-economic or political factors (Cosgrove 2011), looping breaks may arise under the pressure of different non-clinical forces. For instance, mainstream psychiatric narratives derived from the biomedical model, which tend to associate psychiatric categories with fixed biological entities, reinforce rigid diagnostic perceptions, thus restricting the integration of individual testimonies and reinforcing the epistemic asymmetry between clinicians and patients. Epistemic injustices based on the clinician’s role can act as operators of looping breaks, but they are not exclusive. Affirming their existence does not deny the existence of other factors that may also be involved, potentially engaging different forms of injustice (e.g., distributive injustices, such as disparities in funding or public attention between different professional societies, could play a particularly significant role within public or private institutions). The relationship and relative weight of such injustices in connection with epistemic injustice would warrant further investigation in future work. As before, however, our primary focus will be on looping breaks that involve clinicians in their interactions with patients. Without claiming to conduct a sociological investigation, we justify this focus by noting that it consistently involves a chain of actors in more or less direct contact with patients or revision committees (Latour 1988). Accordingly, even when the loop does not directly involve a clinician relying on accumulated experience to transmit it to revision committees, it still involves clinicians who have shared their insights with other individuals (clinicians or not, including for instance, policymakers) who in turn relay messages originating from the patients concerned (Epstein 2009). Of course, as we have described in relation to the looping effect itself, looping breaks may be accounted for by various types of conventions, hidden motivations and invisible colleges, political, economic, social, or pharmaceutical interests (Ghaemi 2009), as well as value-laden stakes—none of which are denied by the analysis that follows. However, we chose not to explore each factor in depth—such as the socioeconomic framing of depression (Sato 2014), social constructionist accounts (Schaffner and Tabb 2015), economic interests and invisible colleges (Blashfield 1982)—as doing so would require a distinct methodological framework and significantly expand the scope of the present work.

9 However, due to the wide diversity of patients with a psychiatric disorder, including all testimonials and their differences in the classification could weaken psychiatric categories (e.g., in terms of reliability, clarity or manipulability). Assuming all intra-class variations would lead to extreme nosographic volatility (Phillips et al. 2012). We take this as a legitimate worry. Nosological stability is a fair demand from clinicians. While we do not have space to engage in a in length discussion over this problem, we believe that a non-ideal solution could be achieved, with a critical balance between nosological stability and epistemic justice. From this nuanced vision could arise a number of questions addressing methodological issues and tensions between population-level and individual-level interests (e.g., “Do population and epidemiological issues related to nosological stability justify epistemic injustices at the individual level?”), themselves likely to lead to collective discussions between patients and clinicians (e.g., professional organizations engage with activists and advocates).
